# Ultrasound-guided stellate ganglion block attenuates early postoperative visceral pain after laparoscopic hysterectomy: A prospective randomized controlled trial

**DOI:** 10.1371/journal.pone.0339677

**Published:** 2025-12-30

**Authors:** Jing Lin, Yue Huang, Yaling Wen, Wanli Yang, Youbo Zuo

**Affiliations:** 1 Department of Anesthesiology, Affiliated Hospital of North Sichuan Medical College, Nanchong, Sichuan, China; 2 Department of Pain Medicine, Mianyang 404 Hospital, Mianyang, Sichuan, China; Government Gousia Hospital, DHS, Srinagar, INDIA

## Abstract

**Background:**

Postoperative visceral pain remains a major challenge following laparoscopic hysterectomy. While stellate ganglion block (SGB) is increasingly utilized for acute and chronic pain management, limited evidence exists regarding its efficacy in modulating visceral pain after gynecologic laparoscopy. This study aimed to evaluate whether ultrasound-guided SGB could reduce early postoperative visceral pain intensity and opioid consumption.

**Methods:**

In this prospective, randomized controlled trial, 90 patients undergoing laparoscopic hysterectomy were allocated (1:1:1) to receive ultrasound-guided SGB combined with transversus abdominis plane block (TAPB) (SGB group), TAPB alone (TAP group), or no nerve block (control group). The primary outcome was visceral pain intensity, assessed using visual analog scale (VAS) scores at rest and during movement at 1, 3, 6, 24, and 48 hours postoperatively. Secondary outcomes included rescue analgesia requirements and complications.

**Results:**

The linear mixed-effects model revealed that the SGB group exhibited a significantly greater reduction in visceral pain intensity at rest and during movement at 1, 3, and 6 hours compared to the TAP and control groups (*P* < 0.05). Notably, the percentage of patients requiring rescue analgesia was significantly lower in the SGB group compared to the TAP and control groups (14.3% vs. 32.1% and 48.1%, respectively, *P* < 0.05). No statistically significant differences in incisional pain were detected among the three groups at any time point (*P* > 0.05).

**Conclusion:**

Ultrasound-guided SGB effectively alleviates early postoperative visceral pain and reduces opioid demand, supporting its role as a valuable addition to multimodal analgesia protocols in laparoscopic hysterectomy.

## Introduction

Laparoscopic hysterectomy has become a common surgical approach for treating various gynecological disorders. Over 600,000 hysterectomies are performed annually in the United States alone, with 60–70% now using laparoscopic approaches [1]. Although minimally invasive, laparoscopic hysterectomy can cause moderate-to-severe postoperative pain [2,3]. Pain associated with laparoscopic gynecological surgery mainly consists of incisional pain and visceral pain [4]. Visceral pain, in particular, can persist for up to 72 hours postoperatively [2]. Currently, TAP block primarily targets incisional pain, leaving postoperative visceral pain management largely dependent on opioids [4,5]. However, the side effects of opioids often limit their use, which may hinder patients’ postoperative recovery and increase the risk of chronic pain [6,7].

Visceral pain is associated with autonomic nerve instability and elevated levels of norepinephrine and inflammatory mediators. Visceral sensation is intricately modulated by autonomic neural inputs, with significant involvement of both parasympathetic and sympathetic pathways [8]. Norepinephrine (NE), which is synthesized and secreted by postganglionic sympathetic neurons and central adrenergic nerve endings, plays a pivotal role in the genesis of postoperative visceral pain [9]. Surgical intervention and tissue inflammation may precipitately heighten the sensitivity of pain-sensing nerves, while the release of inflammatory mediators can augment the transmission of visceral pain signals. A recent trial also demonstrated that SGB could reduce visceral pain after cholecystectomy [10]. The analgesic effect of SGB may stem from its dual modulation of sympathetic hyperactivity and proinflammatory cytokine release. By blocking cervical sympathetic fibers, SGB likely attenuates NE-driven visceral sensitization while suppressing surgical stress-induced elevation of IL-6 and TNF-α, as evidenced in prior studies [11]. In addition, SGB can also accelerate the metabolism of substances such as 5-hydroxytryptamine [12]. Based on these mechanisms, SGB is extensively utilized in managing multiple symptoms and diseases, such as acute and chronic pain, refractory arrhythmia, postoperative discomfort, menopause symptoms, post-traumatic stress disorder (PTSD), long coronavirus disease (COVID) syndrome, intractable hiccups, and excessive daytime sleepiness [13]. In recent years, numerous studies have also utilized SGB for postoperative pain management [14]. However, few randomized controlled trials have evaluated the role of SGB in visceral pain management after laparoscopic hysterectomy.

This randomized controlled trial (RCT) aimed to evaluate the efficacy of ultrasound-guided SGB in visceral pain management after laparoscopic hysterectomy.

## Methods

### Patients

This single-center RCT was approved by the Ethics Committee of the Affiliated Hospital of North Sichuan Medical College (2023ER004−1), and registered on the Chinese Clinical Trial Registry (ChiCTR, http://www.chictr.org.cn) with registration number ChiCTR2300069134. The study was conducted in accordance with the Declaration of Helsinki, and written informed consent was obtained from all participants.

**Fig 1:**
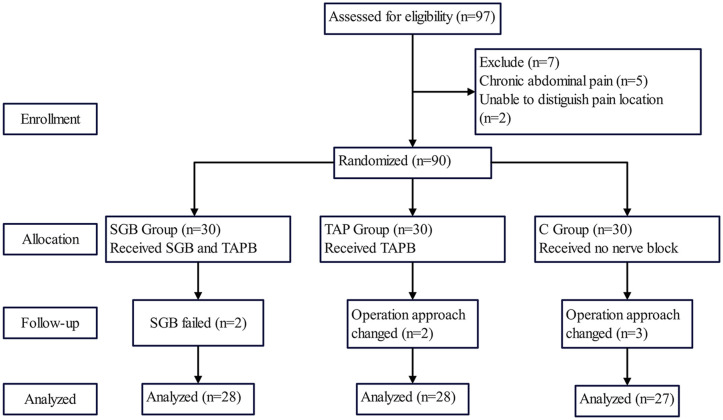
CONSORT diagram of the trial.

A total of 90 patients scheduled for laparoscopic hysterectomy were enrolled from March 9th 2023 to July 30th 2023 at the Affiliated Hospital of North Sichuan Medical College (Fig 1). Inclusion criteria were patients aged 18–65 years, belonging to the American Society of Anesthesiologists (ASA) physical status I to III, body mass index (BMI) between 18.0 and 30.0 kg/m^2^. Exclusion criteria included chronic opioid use (>3 days/week for ≥1 month preoperatively), history of chronic abdominal/pelvic pain, major organ dysfunction (e.g., heart failure, cirrhosis), inability to cooperate with VAS assessment, and allergy to lidocaine or ropivacaine. Failed nerve block (defined below), changed surgical approach (switch from laparoscopic to open hysterectomy) were also excluded.

**Fig 2:**
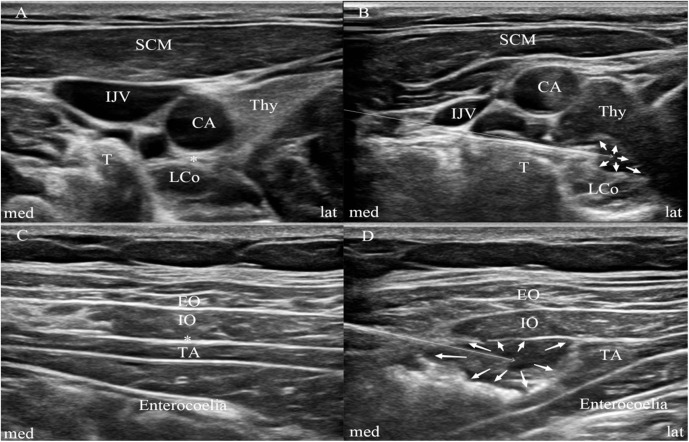
Ultrasound-guided SGB and TAPB. **Notes:** A and B, Sonographic image of SGB (the transverse short-axis view at the C6 level). C and D, Sonographic image of the lateral TAP block. White arrows show the subfascial spread of local anesthetics. Asterisk represents the layer of target. CA, carotid artery; IJV, internal jugular vein; PVF, prevertebral fascia; SCM, sternocleidomastoid muscle; LCo, longus colli; Thy, thyroid gland; T, anterior tubercle of C6 transverse process; EO, external oblique muscle; IO, internal oblique muscle; TA, transversus abdominis muscle; med, medial; lat, lateral.

SGB failure: Ultrasound-confirmed absence of solution spread superficial to the longus colli muscle (Fig 2A-2B) immediately post-injection, or no clinical signs of sympathetic blockade (e.g., no skin warming of the ipsilateral upper extremity) within 15 min.

TAP failure: Ultrasound-confirmed absence of solution spread in the transversus abdominis plane (Fig 2C-2D) immediately post-injection.

### Randomization

Eligible patients were randomly assigned (1:1:1) to three groups on the day before surgery using a computer-generated random number table and sealed envelope method, all envelopes were prepared by an independent statistician not involved in patient recruitment or outcome assessment. The three groups were: (1) SGB group: ultrasound-guided SGB combined with TAP block; (2) TAP group: ultrasound-guided TAP block alone; (3) Control group: no nerve block.

### Intervention and blinding

All nerve blocks were performed by a single experienced anesthesiologist with over two years of clinical practice in ultrasound-guided regional anesthesia. Blocks were administered at the end of surgery (before extubation). The SGB group received ultrasound-guided right-sided SGB using 6 mL of 1% lidocaine and bilateral TAPB with 30 mL of 0.33% ropivacaine, 15 mL per side. The TAP group received ultrasound-guided bilateral TAPB with 30 mL of 0.33% ropivacaine alone, 15 mL per side. The control group received no nerve block.

Blinding was maintained as follows: (1) The anesthesiologist performing the blocks did not participate in follow-up or outcome assessment; (2) Outcome assessors (responsible for VAS scoring and data collection) were physically separated from the operating room, unaware of group allocation, and used standardized scripts for pain assessment; (3) Patients were blinded to their group allocation, being informed only that they might receive one or more regional blocks for pain relief; (4) Patient records were sealed in an envelope until the trial was completed.

### General anesthesia and monitoring

All patients fasted for 8 hours preoperatively without premedication. Standard monitoring included pulse oximetry, electrocardiography and noninvasive blood pressure. Anesthesia was induced with intravenous sufentanil 0.3–0.5 µg/kg, propofol 1.5–2 mg/kg and cisatracurium 0.15 mg/kg. Tracheal intubation was performed 3–5 min after induction.

Anesthesia was maintained with inhalational sevoflurane and intermittent intravenous injection of sufentanil and cisatracurium. Mechanical ventilation was initiated with a tidal volume of 6-8mL/kg and the respiratory rate was adjusted to maintain the end-tidal carbon dioxide partial pressure (PetCO_2_) between 35 and 45 mmHg. Patients in the SGB and TAP groups received ultrasound-guided TAPB with or without SGB after the surgery, while the control group received no regional block. The endotracheal tube was extubated after recovery of spontaneous breathing, adequate tidal volume, swallowing reflex and consciousness. Anesthetic dosage was continuously adjusted to maintain a bispectral index (BIS) value between 40 and 60 and mean arterial pressure within 20% of baseline. Ephedrine was administrated if the blood pressure was more than 20% below the baseline, and atropine was administered if the heart rate was lower than 50 beats per minute.

### Ultrasound-guided block procedures

All blocks were performed by an experienced anesthesiologist using a high-frequency (5–10 MHz) linear ultrasound probe (Mindray M9). A 22 G sterile nerve stimulation needle (0.71 × 80 mm, B. Melsungen, Germany) was used in-plane short-axis technique. The SGB group received ultrasound-guided SGB combined with TAPB, the TAP group received ultrasound-guided TAPB.

### SGB

For the SGB group, a right-sided block was performed using the paratracheal, in-plane approach [15]. The patients were placed in the supine position with thin pads placed beneath both shoulders. After skin sterilization, the C7 level was first identified under ultrasound guidance using a lateral approach to the right interscalene space. The ultrasound probe was then slowly moved cephalad until the C6 level was identified, corresponding to the first transverse process with anterior and posterior tubercles. The needle was inserted from the lateral aspect of the neck, avoiding major nerves and vessels, until the tip reached the C6 transverse process, 6 mL of 1% lidocaine (Shiyao, China) was injected beneath the prevertebral fascia superficial to the longus colli muscle after confirming the absence of blood, gas or cerebrospinal ﬂuid during withdrawing. Vital signs were monitored throughout. Successful SGB was confirmed by doppler imaging of needle-tip placement and visualization of injected solution spreading superficial to the longus colli muscle (Fig 2A-2B).

### TAPB

Ultrasound-guided bilateral TAPB was performed with 30 mL of ropivacaine 0.33% (Ropivacaine, Molteni, Italy) in supine position [16]. For the lateral TAPB, the ultrasound probe was placed in the midaxillary line between the iliac crest and the costal margin to identify the external oblique muscle, internal oblique muscle and transversus abdominis muscle. After sterilization, the needle was inserted using an in-plane technique, when the tip reached the level of the transversus abdominis muscle plane, 15 mL of 0.33% ropivacaine was injected after confirming the absence of blood or gas during withdrawing. The same procedure was repeated on the opposite side. The appearance of a widely dispersed oval-shaped ultrasound image at the transversus abdominis muscle plane indicated a successful injection (Fig 2C-2D).

### Data collection

The anesthesiologist responsible for postoperative data collection was blinded to group allocation and was not present in the operating room during administration of anesthesia or performance of block. The demographic characteristics of the patients, including age, BMI, ASA, menopause, the duration of surgery and anesthesia were recorded.

The day before surgery, the patients were trained to use the visual analogue scale (VAS, ranging from 0 cm [no pain] to 10 cm [worst imaginable pain]) to assess the intensity of acute postoperative pain. Meanwhile, the two main pain components after laparoscopic surgery were explained to the patients in detail. Incisional pain was defined as superficial, well-localized pain in the abdominal wall; visceral pain was defined as pain inside the abdomen, which may be deep, dull, and difficult to localize. The VAS scores for postoperative incision pain and visceral pain were evaluated at rest and movement (cough and deep breathing) respectively at 1, 3, 6, 24, and 48 hours postoperatively to capture the peak and trajectory of early postoperative pain. Each patient was supplied with a questionnaire consisting of VAS score forms. Intramuscular pethidine was administered as rescue analgesic if VAS > 4, the number of doses and total amount were recorded. Other variables were also recorded, including the time of first flatulence and the incidence of postoperative nausea and vomiting (PONV).

### Sample size

The sample size of this study was calculated based on the visceral pain VAS scores at 6 hours postoperatively obtained from a pilot study, 20 patients were assigned to compare the SGB and TAP groups (n = 10). Using the StatBox-online statistical computing system, we compare the VAS scores of the two groups (1.5 ± 1.65 vs 2.7 ± 1.3) with a Student’s t-test. Assuming a type I error (α) of 0.05 and statistical power (1 − β) of 0.80, a sample size of 27 patients per group was required. Accounting for a potential 10% dropout rate, 30 patients per group were enrolled.

### Statistical analysis

All data were analyzed by SPSS 26.0 (IBM Corp., NY, USA). The normal distribution of continuous variables was assessed by the Shapiro-Wilk test, normal distribution were expressed as mean ± SD and use F-test to test for homogeneity of variances and were compared using analysis of variance (ANOVA), for all multiple hypothesis testing, a correction for multiple comparisons using the Bonferroni method was applied to control for Type Ⅰ error inflation, ensuring that significant findings across multiple time points and variables were robust and not due to chance. Non-normally distributed data are presented as median (interquartile range, IQR), with the Kruskal-Wallis H test for overall comparisons; Bonferroni-corrected Mann-Whitney U tests were used for post-hoc pairwise comparisons. Categorical variables were presented as frequencies (n, %) and analyzed using the chi-square test; Fisher’s exact test was used when expected cell frequencies were <5. All tests were two-sided, and a *P* < 0.05 was considered statistically significant.

## Results

### Patient flow and baseline characteristics

From March 9 to July 30, 2023, 97 patients were screened in our trial, 7 patients were excluded (5 with chronic abdominal pain, 2 unable to complete questionnaires). The remaining 90 patients were randomly assigned to the SGB, TAP, and control groups (n = 30 each). Seven patients were lost to follow-up, resulting in 83 patients for final analysis (Fig 1). There were no significant differences among the three groups in terms of demographic and intraoperative characteristics including age, BMI, ASA, menopause, duration of surgery, anesthesia time, fluid infusion, blood loss, and sufentanil consumption (*P* > 0.05, Table 1).

**Table 1:** Demographic and intraoperative characteristics.

Variables	SGB group (n = 28)	TAP group (n = 28)	Control group (n = 27)	*P* value
Age (years)	49.6 ± 4.7	51.9 ± 5.4	51.1 ± 5.2	0.186
BMI (kg/m2)	23.0 ± 2.3	24.0 ± 2.2	24.2 ± 1.9	0.069
ASA (Ⅱ/Ⅲ, n)	24/4	21/7	22/5	0.592
Menopause (Yes/No, n)	12/18	18/12	9/16	0.149
Duration of surgery (min)	133.5 (68.3,159.3)	145 (92.5,178.8)	127 (105,170)	0.803
Anesthesia time (min)	180 (136.3,205)	180 (135,215)	180 (160,225)	0.778
Sufentanil consumption (ug)	45.0 (40.0,50.0)	50.0 (45.0,58.8)	50.0 (45.0,60.0)	0.162
Total fluid infusion (ml)	1600 (1400,1775)	1600 (1400,2175)	1600 (1600,2100)	0.155
Blood loss (ml)	50.0 (50.0-100.0)	50.0 (30.0-100.0)	80.0 (40.0-200.0)	0.577

**Notes:** Variables are expressed as mean ± SD, number or Median (interquartile range) as appropriated. **Abbreviations:** BMI, body mass index; ASA, American Society of Anesthesiology.

### Postoperative pain scores

**Fig 3:**
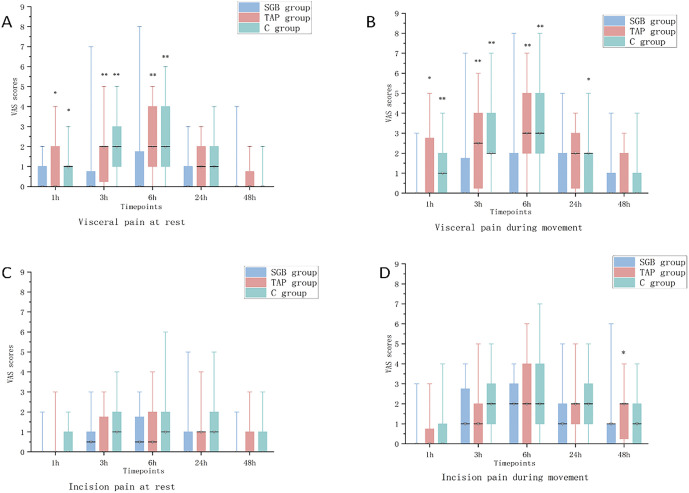
VAS scores for all time points. **Notes: A.** Visceral pain when resting. **B.** Visceral pain when moving. **C.** Incision pain when resting. **D.** Incision pain when moving. SGB group, SGB and lateral TAPB. TAP group, lateral TAPB. C group (control group), no nerve block. *P < 0.05, SGB group vs TAP group and control group; **P < 0.01, SGB group vs TAP group and control group.

The SGB group had significantly lower visceral pain VAS scores at rest and during movement compared to the TAP and control groups at 1, 3, and 6 hours postoperatively (*P* < 0.05, Fig 3A-3B). No significant differences were observed at 24 and 48 hours (*P* > 0.05). No significant differences in incisional pain VAS scores were detected among the three groups at any time point (*P* > 0.05, Fig 3C-3D).

### Postoperative analgesic consumption

The percentage of patients requiring rescue analgesia was significantly lower in the SGB group compared to the TAP and control groups (14.3% vs. 32.1% and 48.1%, respectively, *P* < 0.05), as well as a lower total dose of pethidine consumed (*P* < 0.05, Table 2).

**Table 2:** Cumulative number of rescue analgesic and postoperative pethidine consumption.

Variables	SGB group (n = 28)	TAP group (n = 28)	Control group (n = 27)	*P* value
Cumulative number of rescue analgesic (n)				0.025
None	23	15	16	
Once	4	8	11	
Twice	0	1	2	
Postoperative pethidine consumption (n)	4	9	13	0.025

**Notes:** Data are presented as number of patients.

### Postoperative recovery outcomes

No significant differences were found in postoperative recovery indicators, including PONV, first flatulence after surgery, and length of postoperative hospital stay (*P *> 0.05, Table 3). No severe complications were reported, minor complications related to SGB included transient Horner syndrome, which was observed in 2 patients and resolved spontaneously within 24 hours. No hoarseness or TAP block-related complications (e.g., peritoneal puncture, local hematoma) were observed (*P* > 0.05, Table 3).

**Table 3:** Incidence of complications during the first two postoperative days.

Variables	SGB group (n = 28)	TAP group (n = 28)	Control group (n = 27)	*P* value
PONV (Yes/No, n)	16/12	20/8	21/6	0.238
First flatus after surgery (h)	33.0 (29.5-38.2)	35.5 (28.6-39.0)	34.0 (30.0-38.0)	0.856
Postoperative hospital stay (d)	6.0 (5.0-7.8)	6.0 (6.0-8.8)	6.0 (5.0-7.0)	0.222
SGB-Related complications, n (%)				
Transient horner syndrome	2 (7.1)	0 (0)	0 (0)	0.083
Hoarseness	0 (0)	0 (0)	0 (0)	1.000
TAP-related complications, n (%)				
Local hematoma	0 (0)	0 (0)	0 (0)	1.000
Peritoneal puncture	0 (0)	0 (0)	0 (0)	1.000

**Notes:** Data are presented as median (interquartile range) or number of patients. **Abbreviations:** PONV, postoperative nausea and vomiting.

## Discussion

In summary, our study found that ultrasound-guided SGB effectively suppresses early postoperative visceral pain and reduces analgesic consumption after laparoscopic hysterectomy, but has no effect on incisional pain.

Laparoscopic hysterectomy is a minimally invasive gynecological surgery, that significantly reduces surgical trauma from the incision. Local incisional infiltration and peripheral nerve blocks further effectively alleviated incision pain of laparoscopic abdominal surgery [16]. However, visceral pain remains difficult to control after laparoscopic gynecological surgery [4]. In our study, the VAS of incision pain and visceral pain, the times and dose of rescue analgesic showed no difference between the TAP and C groups, which also indicated that incision pain is not the main problem of laparoscopic hysterectomy [17]. This finding is consistent with SGB’s mechanism of action that SGB modulates sympathetic pathways involved in visceral pain, not the somatic sensory nerves. Previous studies have reported that SGB can play an analgesic role in early postoperative pain [5,18]. Those results confirmed that SGB could effectively inhibit visceral pain for 24 hours after laparoscopic hysterectomy and reduce the consumption of postoperative analgesics, this is consistent with our study. The reduced postoperative pethidine consumption in the SGB group underscores its clinical relevance, as opioid-sparing effects are strongly associated with decreased risks of opioid-related adverse events (e.g., respiratory depression, ileus) and enhanced recovery [19]. Although the cumulative rescue analgesia count showed only a marginal trend (*P *= 0.098), this may reflect insufficient statistical power due to the small sample size or variability in individual pain thresholds. Notably, the lack of significant differences in shoulder or incisional pain across groups further supports the specificity of SGB in targeting visceral pain mechanisms rather than generalized analgesia.

Laparoscopic surgery trauma and CO_2_ pneumoperitoneum can lead to increased sympathetic nerve activity, imbalance of sympathetic and parasympathetic branches of autonomic nervous system, and increased NE synthesis and secretion of postganglionic neurons of sympathetic nerve and adrenergic nerve endings in brain, resulting in stress-related internal environment disorder and pain [9]. Postoperative visceral pain may originate from pneumoperitoneum-induced diaphragmatic irritation and CO₂ retention, as observed in 71% of laparoscopic cases, and the operation of internal tissues during surgery may lead to local tissue damage and trigger inflammation [20]. Various inflammatory mediators such as cytokines, growth factors, and prostaglandins are released at the site of tissue injury, which can activate the surrounding sensory nerve endings and cause pain [11]. In addition, surgical procedures may affect the blood supply to internal organs, leading to ischemia. Ischemia can not only directly damage tissues, but also activate pain receptors by releasing pain sensitizing substances such as lactic acid and ATP [21]. Visceral pain is associated with autonomic nerve instability, elevated inflammatory factors and ischemia of local visceral organs.

To our knowledge, this is the first RCT evaluating SGB in gynecologic laparoscopy, extending previous cholecystectomy findings [11]. SGB inhibits the excitability of the sympathetic-adrenal system. Blood NE levels decrease significantly after SGB in surgical patients but not in healthy individuals, indicating that SGB primarily modulates pathologically increased sympathetic activity to restore autonomic balance [22,23]. This is why our trial chose to perform ultrasound guided SGB at the end of surgery. Zhu et al. [13] reported that SGB promoted the recovery of gastrointestinal function in patients undergoing laparoscopic colorectal surgery, alleviated stress response, and reduced the levels of NE, cortisol, interleukin-6 (IL-6) and C-reactive protein (CRP). Our study demonstrates that SGB significantly reduces early postoperative visceral pain and opioid requirements compared to TAP block and conventional analgesia in patients undergoing gynecological surgery. The superior analgesic efficacy of SGB at 1, 3, and 6 hours postoperatively, both at rest and during movement, aligns with the hypothesis that visceral pain is predominantly mediated by sympathetic pathways [21]. By modulating sympathetic tone and attenuating nociceptive signaling from visceral afferents, SGB may disrupt central sensitization and reduce pain intensity during the critical early postoperative period [15,21]. In contrast, TAP block primarily targets somatic pain pathways via blockade of abdominal wall sensory nerves, which explains its limited efficacy in alleviating visceral pain [24]. These findings are consistent with prior studies which emphasized the distinct neuroanatomical pathways of visceral versus somatic pain [25].

There were no significant differences in postoperative incisional pain among the three groups, this may be the incisional pain was not the main complaint after the minimally invasive gynecological surgery. Choi et al. [3] also reported visceral pain dominated over incisional pain constantly for 72 hours after total laparoscopic hysterectomy. The overall incidence of PONV was high in three groups, but the incidence of postoperative PONV was lower in the SGB group (57.1%) compared to the TAP group (71.4%) and the control group (77.8%), primarily attributed to reduced postoperative opioid utilization and diminished opioid-related adverse effects, this may be attributed to several risk factors in this population, including female gender, laparoscopic gynecological surgery, use of volatile anesthesia, and non-smoking status [26]. Furthermore, a substantial body of literature has documented the beneficial impact of SGB on gastrointestinal tract recovery in surgical patients [13,27]. Therefore, we hypothesize that SGB might mitigate PONV. Although not statistically significant, the time to first flatus was numerically shorter in the SGB group. SGB could reduce opioid requirements, but its limited impact on recovery metrics highlights the need for multimodal strategies integrating pain management, early enteral nutrition, and physiotherapy to optimize postoperative rehabilitation, further rigorous clinical trials are warranted to substantiate these findings [28].

We considered that Horner syndrome, which can be caused by SGB, might increase the discomfort of patients, so a low concentration and small volume of lidocaine were used. Due to the influence of anatomical factors, the right stellate ganglion block is less likely to damage other organs and blood vessels, so we choose the right side block [29].

### Limitations

The study has several limitations. First, the single-center and modest sample size may limit generalizability, particularly regarding rare complications, and the results require confirmation by further research. Second, the absence of inflammatory biomarker analysis precludes definitive mechanistic conclusions, future biomarker-embedded studies should verify this immunomodulatory pathway in gynecologic populations. Third, The 48-hour follow-up window precludes evaluation of long-term outcomes like chronic postsurgical pain (CPSP) development. Future multicenter trials with larger cohorts and extended follow-up are needed to validate these findings and explore SGB’s potential synergistic effects with enhanced recovery after surgery (ERAS) protocols.

### Conclusion

In summary, our study found that ultrasound-guided SGB could effectively suppress visceral pain in the early postoperative period of laparoscopic hysterectomy and reduce the postoperative analgesic consumption. However, SGB has no effects on incisional pain after surgery.

## Supporting information

S1 File Clinical trial protocol. (PDF)

S2 File CONSORT checklist. (DOCX)

S3 File Data of study. (XLS)

S4 File Clinical trial protocol-chinese. (PDF)

## References

[1] Plante M, Mahner S, Sebastianelli A, Bessette P, Lambaudie E, Guyon F, et al. Minimally invasive compared to open surgery in patients with low-risk cervical cancer following simple hysterectomy: an exploratory analysis from the Gynegologic Cancer Intergroup/Canadian Cancer Trials Group CX.5/SHAPE trial. Int J Gynecol Cancer. 2025;35(1):100001. doi: 10.1016/j.ijgc.2024.100001 39878257

[2] Qi M, Xiao W, Yang S, Wang S, Zhou L, Wan A, et al. Pre-incisional preventive precise multimodal analgesia may enhance the rehabilitation process of acute postoperative pain following total laparoscopic hysterectomy: a randomized controlled trial. Pain Physician. 2023;26(3):E123–31. doi: 10.36076/ppj.2023.26.e123 37192230

[3] Choi JB, Kang K, Song MK, Seok S, Kim YH, Kim JE. Pain characteristics after total laparoscopic hysterectomy. Int J Med Sci. 2016;13(8):562–8. doi: 10.7150/ijms.15875 27499688 PMC4974904

[4] Yang G-W, Cheng H, Song X-Y, Yang Y-F, Liu H, Ji F-H, et al. Effect of oxycodone-based multimodal analgesia on visceral pain after major laparoscopic gastrointestinal surgery: a randomised, double-blind, controlled trial. Drug Des Devel Ther. 2024;18:1799–810. doi: 10.2147/DDDT.S464518 38828025 PMC11141770

[5] Raina J, Costello C, Suarthana E, Tulandi T. Postoperative discharge opioid consumption, leftover, and disposal after obstetric and gynecologic procedures: a systematic review. J Minim Invasive Gynecol. 2022;29(7):823-831.e7. doi: 10.1016/j.jmig.2022.04.017 35513302

[6] Covotta M, Claroni C, Costantini M, Torregiani G, Pelagalli L, Zinilli A, et al. The effects of ultrasound-guided transversus abdominis plane block on acute and chronic postsurgical pain after robotic partial nephrectomy: a prospective randomized clinical trial. Pain Med. 2020;21(2):378–86. doi: 10.1093/pm/pnz214 31504875

[7] Wu Q, Zhou Y, Sun S, Li H, Cao S, Shou H. Clinical analysis of acute postoperative pain after total laparoscopic hysterectomy for adenomyosis and uterine fibroids - a prospective observational study. Ann Med. 2023;55(2):2281510. doi: 10.1080/07853890.2023.2281510 37994446 PMC10836289

[8] Vorenkamp K, Yi P, Kemp A. Sympathetic Blocks for Visceral Pain. Phys Med Rehabil Clin N Am. 2022;33(2):475–87. doi: 10.1016/j.pmr.2022.01.010 35526980

[9] Rahimzadeh P, Mahmoudi K, Khodaverdi M, Faiz SHR. Effects of ultrasound guided ganglion stellate blockade on intraoperative and postoperative hemodynamic responses in laparoscopic gynecologic surgery. Wideochir Inne Tech Maloinwazyjne. 2020;15(2):351–7. doi: 10.5114/wiitm.2019.89653 32489497 PMC7233162

[10] Methods In Medicine CAM. Retracted: effect of stellate ganglion block combined with lidocaine at different concentrations for preemptive analgesia on postoperative pain relief and adverse reactions of patients undergoing laparoscopic cholecystectomy. Comput Math Methods Med. 2022;2022:9804601. doi: 10.1155/2022/9804601 36466555 PMC9715313

[11] Li Y, Loshak H. Stellate ganglion block for the treatment of post-traumatic stress disorder, depression, and anxiety. Ottawa (ON): Canadian Agency for Drugs and Technologies in Health; 2021. 34255448

[12] Zhu G, Kang Z, Chen Y, Zeng J, Su C, Li S. Ultrasound-guided stellate ganglion block alleviates stress responses and promotes recovery of gastrointestinal function in patients. Dig Liver Dis. 2021;53(5):581–6. doi: 10.1016/j.dld.2020.11.028 33303314

[13] Ren Y, Zhang Z, Li H-P, Zhang P-J, Duo J, Kong H. A comprehensive overview of the stellate ganglion block throughout the past three decades: a bibliometric analysis. Pain Physician. 2024;27(5):E597–610. doi: 10.36076/ppj.2024.7.e597 39087973

[14] Xiang X-B, Wu Y-Y, Fang Z, Tang X, Wu Y-L, Zhou J, et al. Stellate ganglion block for visceral pain in elderly patients undergoing video-assisted thoracoscopic lung cancer surgery: a randomized, controlled trial. Int J Surg. 2024;110(11):6996–7002. doi: 10.1097/JS9.0000000000001867 38913440 PMC11573066

[15] Pinarbaşi A, Altiparmak B, Korkmaz Toker M, Pirinççi F, Uğur B. Ultrasound-guided transversalis fascia plane block or transversus abdominis plane block for recovery after caesarean section: a randomised clinical trial. Eur J Anaesthesiol. 2024;41(10):769–78. doi: 10.1097/EJA.0000000000002041 39039833

[16] Wageh M, Sultan MA, Moawad HES, Mokbel EM, Alseoudy MM. Ultrasound guided quadratus lumborum block versus interlaminar epidural block for analgesia in pediatric abdominal surgery: a randomized controlled trial. BMC Anesthesiol. 2024;24(1):180. doi: 10.1186/s12871-024-02548-z 38773360 PMC11107015

[17] Schnabel A, Carstensen VA, Lohmöller K, Vilz TO, Willis MA, Weibel S, et al. Perioperative pain management with regional analgesia techniques for visceral cancer surgery: a systematic review and meta-analysis. J Clin Anesth. 2024;95:111438. doi: 10.1016/j.jclinane.2024.111438 38484505

[18] Hirai R, Uesawa Y. Analysis of opioid-related adverse events in Japan using FAERS database. Pharmaceuticals (Basel). 2023;16(11):1541. doi: 10.3390/ph16111541 38004407 PMC10675800

[19] Ruiz-Tovar J, Garcia A, Ferrigni C, Duran M. Application of Vitamin E acetate on staple lines and anastomoses of Roux-en-Y gastric bypass: impact on postoperative pain and acute phase reactants. Obes Surg. 2020;30(8):2988–93. doi: 10.1007/s11695-020-04635-9 32342266

[20] Yin M, Li Z-H, Wang C, Li Y, Zhang H, Du H-B, et al. Stellate ganglion blockade repairs intestinal mucosal barrier through suppression of endoplasmic reticulum stress following hemorrhagic shock. Int J Med Sci. 2020;17(14):2147–54. doi: 10.7150/ijms.47662 32922175 PMC7484657

[21] Lee M-C, Bartuska A, Chen J, Kim RK, Jaradeh S, Mihm F. Stellate ganglion block catheter for paroxysmal sympathetic hyperactivity: calming the “neuro-storm”. Reg Anesth Pain Med. 2023;48(10):522–5. doi: 10.1136/rapm-2023-104399 37230754

[22] Geng J, Wang J, Zhang Y, Song W, Zhu J, Chen J, et al. The effect of a combined modified pectoral and stellate ganglion block on stress and inflammatory response in patients undergoing modified radical mastectomy. Int J Breast Cancer. 2022;2022:3359130. doi: 10.1155/2022/3359130 35707316 PMC9192316

[23] Lee M-C, Bartuska A, Chen J, Kim RK, Jaradeh S, Mihm F. Stellate ganglion block catheter for paroxysmal sympathetic hyperactivity: calming the “neuro-storm”. Reg Anesth Pain Med. 2023;48(10):522–5. doi: 10.1136/rapm-2023-104399 37230754

[24] Boezaart AP, Smith CR, Chembrovich S, Zasimovich Y, Server A, Morgan G, et al. Visceral versus somatic pain: an educational review of anatomy and clinical implications. Reg Anesth Pain Med. 2021;46(7):629–36. doi: 10.1136/rapm-2020-102084 34145074

[25] Kienbaum P, Schaefer MS, Weibel S, Schlesinger T, Meybohm P, Eberhart LH, et al. Update on PONV-What is new in prophylaxis and treatment of postoperative nausea and vomiting? : summary of recent consensus recommendations and Cochrane reviews on prophylaxis and treatment of postoperative nausea and vomiting. Anaesthesist. 2022;71(2):123–8. doi: 10.1007/s00101-021-01045-z 34596699

[26] Yang X, Wu Q, Wang H, Zhang Y, Peng X, Chen L. Effects of ultrasound-guided stellate ganglion block on postoperative quality of recovery in patients undergoing breast cancer surgery: a randomized controlled clinical trial. J Healthc Eng. 2022;2022:7628183. doi: 10.1155/2022/7628183 36046011 PMC9424037

[27] Gan TJ, Belani KG, Bergese S, Chung F, Diemunsch P, Habib AS, et al. Fourth consensus guidelines for the management of postoperative nausea and vomiting. Anesth Analg. 2020;131(2):411–48. doi: 10.1213/ANE.0000000000004833 32467512

[28] Fan Z, Zheng X, Li D, Chen H, Li L. Comparison of lidocaine and ropivacaine stellate ganglion blockade in treating upper limb postherpetic neuralgia. Medicine (Baltimore). 2022;101(23):e29394. doi: 10.1097/MD.0000000000029394 35687777 PMC9276270

[29] Jiang W, Yang L, Zheng X. A prospective randomized clinical trial on the differential effect of left versus right stellate ganglion block on perioperative stress response. BMC Anesthesiol. 2025;25(1):380. https://doi.org/10.1186/s12871-025-03272-y 40739539 10.1186/s12871-025-03272-y PMC12312597

