# Supplementary material for: Ultrasound-guided stellate ganglion block attenuates early postoperative visceral pain after laparoscopic hysterectomy: A prospective randomized controlled trial
Source: PLoS One. 2025 Dec 30;20(12):e0339677. doi: 10.1371/journal.pone.0339677 (PMC12752976; doi:10.1371/journal.pone.0339677)

Effect of Ultrasound-Guided Stellate Ganglion Block on Postoperative Visceral Pain After Laparoscopic Gynecological Surgery: A Prospective Randomized Controlled Study

Version: V1.0

Date: November 10, 2022

Study Duration: December 2022 – October 2023

Study Objective: To investigate whether stellate ganglion block (SGB) improves postoperative visceral pain in patients undergoing laparoscopic gynecological surgery.

Study Design: Randomized Controlled Trial (RCT)

Methodology:

Inclusion Criteria:

1. Patients scheduled for elective laparoscopic gynecological surgery at our hospital.
2. Age between 18 and 65 years.
3. American Society of Anesthesiologists (ASA) physical status classification I-III.
4. Body Mass Index (BMI) between 18 kg/m^2 and 30 kg/m^2.
5. Ability to comprehend and provide written informed consent.

Exclusion Criteria:

1. History of chronic opioid use.
2. History of chronic abdominal and/or pelvic pain.
3. Concomitant severe cerebral, cardiac, pulmonary, hepatic, or renal disease.
4. Inability to cooperate or complete study questionnaires.
5. Allergy to any medication used in the study.

Withdrawal Criteria:

1. Inability to complete surgery laparoscopically, requiring conversion to laparotomy.
2. Failure of the stellate ganglion block or transversus abdominis plane block.

Study Procedures:

Preoperative Day: A researcher will visit the patient to obtain informed consent and provide preoperative instructions (fasting for 8 hours, clear fluids for 6 hours). Patients will be educated on distinguishing postoperative lower abdominal pain types (incisional vs. visceral pain) and instructed on using the Visual Analogue Scale (VAS) for pain intensity scoring. A complete randomization design will be employed: 90 numbers will be randomly assigned to one of three groups. The group assignment and whether SGB is performed will be written on the back of the card corresponding to each number. Cards will be sealed in opaque envelopes. Upon entering the operating room, a nurse will randomly select an envelope to determine the patient's group assignment and intervention.

1 Hour Preoperatively: A research assistant will deliver the sealed envelope to the anesthesiologist. The anesthesiologist will open the envelope to confirm the patient's group assignment and prepare the corresponding anesthetic drugs.

Operating Room Preparation: Upon arrival, standard monitoring (non-invasive blood pressure - NIBP, heart rate - HR, electrocardiogram - ECG, pulse oximetry - SpO_2) will be initiated. The patient will receive supplemental oxygen via face mask, an intravenous line will be established, and the patient will be positioned supine.

Anesthesia Induction: Propofol (1.5-2 mg/kg); Sufentanil (0.3-0.5 μg/kg); Cisatracurium Besylate (0.15 mg/kg). Tracheal intubation will be performed after drug onset. Post-intubation, mechanical ventilation will commence with the following parameters: tidal volume 6-8 mL/kg (based on ideal body weight - IBW), respiratory rate 12-16 breaths/min, inspiratory:expiratory ratio 1:2, inspired oxygen fraction (FiO_2) 40-60%, positive end-expiratory pressure (PEEP) 5-10 cmH_2O, end-tidal CO_2 ($EtCO_2$) target 35-45 mmHg.

Anesthesia Maintenance: FiO_2 40-60%, inhaled sevoflurane 1-3%. Sufentanil and cisatracurium will be administered intermittently to maintain intraoperative blood pressure and heart rate within 20% of baseline values. Anesthesia depth will be monitored using the Bispectral Index (BIS), targeting values between 40-60.

Intraoperative Management: Adverse events (e.g., severe hypertension/hypotension, significant arrhythmia, electrolyte imbalance) will be recorded and managed promptly by an experienced anesthesiologist to maintain stable vital signs (±20% of baseline). Pneumoperitoneum will be established with an initial pressure limit of 14 mmHg and maintained at approximately 12 mmHg.

End of Surgery: Cisatracurium administration will cease 30 minutes before the anticipated end of surgery. Upon surgical completion, the assigned intervention (see below) will be performed. Sevoflurane will then be discontinued, and the lungs will be flushed with 100% oxygen. The tracheal tube will be removed once spontaneous breathing is adequate (satisfactory tidal volume), and protective airway reflexes (swallowing) and consciousness are regained. The patient will be transferred to the

Post-Anesthesia Care Unit (PACU).

PACU: Patients will receive standard PACU monitoring and supplemental oxygen via nasal cannula. The VAS pain score (assessing both incisional and visceral pain) and other parameters will be recorded at 1 hour postoperatively.

Interventions: (Performed immediately upon surgical completion)

SGB Group: Ultrasound-guided right-sided stellate ganglion block (SGB) with 6 ml of 1.0% lidocaine PLUS ultrasound-guided bilateral lateral approach transversus abdominis plane (TAP) block with 30 ml of 0.33% ropivacaine (15 ml per side).

TAP Group: Ultrasound-guided bilateral lateral approach TAP block with 30 ml of 0.33% ropivacaine (15 ml per side).

Control Group (No Intervention Group): No specific block performed.

Ultrasound-Guided Stellate Ganglion Block (SGB)

The patient will be positioned supine with the head turned slightly to the left and a thin pillow under the right shoulder. After standard skin disinfection, a high-frequency linear ultrasound probe (5–10 MHz, Mindray M9) will be placed transversely on the right supraclavicular region, lateral to the trachea. The C6 (carotid artery/jugular vein, thyroid, trachea, longus colli muscle, prevertebral fascia, C6 transverse process) or C7 (carotid artery/jugular vein, vertebral vessels, thyroid, trachea, longus colli muscle, prevertebral fascia, C7 transverse process) level will be identified. Using an in-plane technique with a lateral-to-medial approach (lateral to the carotid sheath, traversing the anterior scalene muscle), the needle will be advanced through the prevertebral fascia. After negative aspiration, 6 ml of 1% lidocaine will be deposited on the surface

of the longus colli muscle. Successful SGB Criteria: Development of Horner's syndrome on the right side: ptosis, miosis, and facial vasodilation (flushing, increased skin temperature, nasal congestion).

Ultrasound-Guided Lateral Approach Transversus Abdominis Plane Block (TAP)

The patient will be positioned supine. The ultrasound probe will be placed transversely in the mid-axillary line, between the iliac crest and the costal margin, to obtain an optimal view of the abdominal wall muscle layers (external oblique, internal oblique, transversus abdominis). The probe will be stabilized transversely just above the iliac crest in the anterior axillary line. The needle will be inserted in-plane, perpendicular to the ultrasound beam at the level of the mid-axillary line. The needle tip will be advanced until positioned within the fascial plane between the internal oblique and transversus abdominis muscles. After negative aspiration for blood or gas, the local anesthetic will be injected. If injection occurs intramuscularly, the needle position will be adjusted before continuing. The procedure will be repeated on the contralateral side. Successful TAP Criteria: Observation of a hypoechoic, elliptical fluid spread within the transversus abdominis plane on ultrasound imaging.

Outcome Measures:

Primary Outcomes: VAS pain scores (assessing incisional pain and visceral pain separately) at 1h, 3h, 6h, 24h, and 48h postoperatively.

Secondary Outcomes:

Total intraoperative sufentanil consumption (μg), postoperative rescue analgesic usage (type, dose, time), time to first ambulation (hours postoperatively), time to first

postoperative flatus (hours postoperatively), postoperative hospital length of stay (days).

Data Management and Analysis: Collected data will be compiled and subjected to statistical analysis. Results will be discussed, and conclusions drawn.

Standardization: All surgical procedures, anesthetic and analgesic management, and perioperative care will be provided by the same medical team.

Visual Analogue Scale (VAS) Definition: ranging from 0 cm [no pain] to 10 cm [worst imaginable pain]

0 = No pain, 1-3 = Mild pain, 4-6 = Moderate pain, 7-10 = Severe pain.

CONSORT diagram of the trial

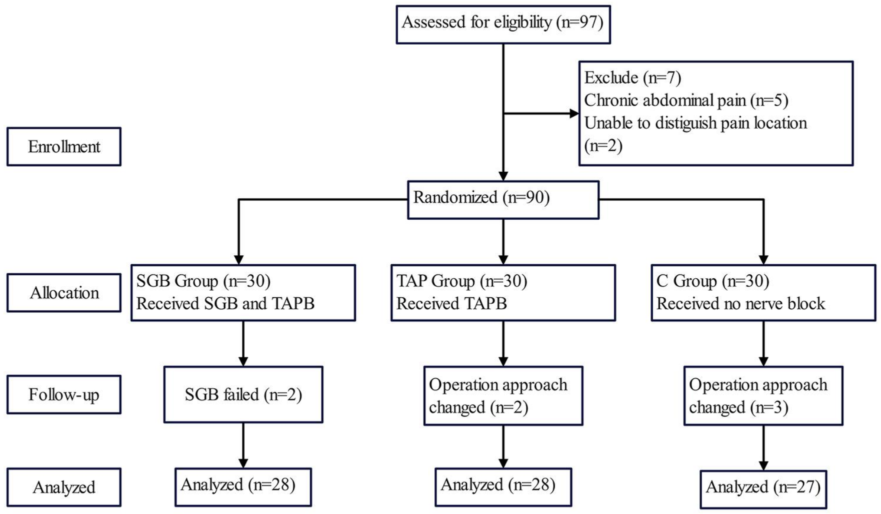

Supplement: S1 File — (PDF) [file pone.0339677.s001.pdf]
