# Supplementary material for: Ultrasound-guided stellate ganglion block attenuates early postoperative visceral pain after laparoscopic hysterectomy: A prospective randomized controlled trial
Source: PLoS One. 2025 Dec 30;20(12):e0339677. doi: 10.1371/journal.pone.0339677 (PMC12752976; doi:10.1371/journal.pone.0339677)
Supplement: S4 File — (PDF) [file pone.0339677.s004.pdf]

超声引导下星状神经节阻滞对腹腔镜妇科手术术后内脏痛的影响：一项前瞻性随机对照研究

版本号：V1.0

版本日期：2022 年 11 月 10 日

研究年限：2022 年 12 月－ 2023 年 10 月

一、研究目的：探讨星状神经节阻滞是否可以改善腹腔镜妇科手术术后内脏痛

二、研究设计：随机对照试验

三、研究方法：

纳入标准：

1.我院择期行“腹腔镜妇科手术”的患者

2.年龄18～65岁；

3.ASA：I-III；

4.$18\ kg/m^2 < BMI < 30\ kg/m^2$

5.能理解并签署书面知情同意书；

排除标准：

1.长期阿片类药物用药史；

2.慢性腹部和/或盆腔疼痛病史

3.合并严重脑、心、肺、肝、肾脏疾病

4.无法配合或完成研究问卷；

5.对研究中使用的药物过敏。

退出标准：

1.腔镜无法完成，术中中转开腹者；

2.星状神经节阻滞或腹横肌平面阻滞无效者

四、研究内容

1.术前一天：由研究者访视患者并签署知情同意书，并交代术前禁食 8 h、禁饮 6h。对患者进行指导，以区分下腹部的术后疼痛，包括切口疼痛和内脏疼痛，患者还接受了 VAS 疼痛强度评分。本实验拟采用完全随机设计方法，90 个号码先随机分为三组，将每个号码对应组是否行星状神经节阻滞写于该编号卡片背面，并将卡片密封于不透光的信封里。患者进入手术室时护士随机抽取一个信封，根

据信封里卡片提示进行操作。

2.术前 1h：研究助理将信封寄送给麻醉医生由麻醉医生拆解信封明确患者分组及治疗方案。准备相应麻醉药品。

3.入室准备：入室后常规监测 NIBP、HR、ECG、SpO2，持续面罩给氧，建立静脉通路，患者取仰卧位。

麻醉诱导：丙泊酚 1.5-2mg/kg；舒芬太尼：0.3-0.5ug /kg；苯磺顺阿曲库铵：0.15mg/kg，待药物起效后进行插管，插管后：机械通气（通气参数：潮气量 6～8mL/kg: IBW，呼吸频率 12～16 次/min，吸呼比 1/2，Fi：40~60%，PEEP:5~10cmH2O,EtCO2:35-45mmHg）。

麻醉维持：氧浓度（FiO2）:40-60%吸入七氟烷 1-3%；间断追加舒芬太尼和顺阿曲库铵，使术中血压心率波动范围维持在基础血压心率的 20%以内，维持根据 BIS 值 40-60。术中管理：记录不良事件，如：严重的高血压或低血压，严重的心律失常，电解质紊乱等，由经验丰富的麻醉医生及时处理，维持患者生命体征平稳（基础值的 20%以内）。术中气腹建立：建立气腹时设置气腹压上限为 14mmHg，术中维持气腹压 12mmhg 左右。

手术结束前：手术结束前 30min 停用注射用苯磺顺阿曲库铵，手术结束时根据分组进行干预，干预结束后停用七氟烷并予以纯氧洗肺。待患者自主呼吸恢复、潮气量满意，吞咽反射及意识恢复后拔除气管导管送入 PACU，患者在 PACU 接受标准监测和经鼻吸氧。并记录 1h 时 VAS 问卷疼痛评分（伤口痛及内脏疼痛）以及其它指标

4.干预措施：

试验组：手术结束后即刻行超声引导下 1.0％利多卡因 6ml 行右侧星状神经节阻滞复合 0.33%罗哌卡因 30ml 双侧侧方入路腹横肌平面神经阻滞组（SGB 组）；

对照组：手术结束后即刻行超声引导下 0.33%罗哌卡因 30ml 双侧侧方入路腹横肌平面神经阻滞组（TAP 组）；

对照组：不做处理。

超声引导星状神经节阻滞（stellate ganglion block, SGB)：

患者取仰卧位，脸转侧向左边，右肩下垫薄枕，常规消毒，采用彩色多普勒超声仪(Mindray M9) 进行引导，探头频率 5–10 MHz，超声探头横 向放置在右

锁骨上气管旁，识别 C6：颈动静脉、甲状腺、气管、颈长肌、颈长肌前筋膜及 C6 横突等结构或识别 C7：颈动静脉、椎血管、甲状腺、气管、颈长肌、颈长肌前筋膜及 C7 横突等结构，采用平面内技 术从外侧入路(颈动脉鞘外侧经前斜角肌) 进针，穿过颈长肌前筋膜，在颈长肌表面注射 1%利多卡因 6ml。SGB 成功的标准为右侧出现霍纳综合征: 右侧出现上睑下垂，瞳孔缩小，颜面管舒张(面红、皮温升高和鼻塞等)。

超声引导侧方入路腹横肌平面阻滞（transversus abdominis plane, TAP）：

患者平卧水平，将超声探头放入腋中线、髂嵴和肋缘之间,取超声显示器上出现最佳腹壁肌肉结构的皮肤位置。此时超声探头横向固定在腋前线髂嵴上方，穿刺针垂直腋中线水平，以平面内技术进针，当针尖显示到达腹横肌平面时，回抽无血或气体时，即可注入一定量的局麻药。若发现药液在肌肉内扩散则需停止注射调整针尖位置,使针尖位于腹横筋膜平面内。在另一边重复同样的操作。当在腹横肌平面层出现梭形广散的超声影像时表示注射成功。

5.观察指标：

主要指标： 术后 1h、3h、6h、24h、48hVAS 评分（包括内脏痛和切口痛）

次要：舒芬太尼总量，术后补救镇痛药使用情况，术后第一次下床时间，术后第一次排气时间，术后住院时间，

6.整理数据资料，并对数据进行统计分析。对分析结果进行讨论并得出结论。

所有手术过程、麻醉和镇痛管理以及围手术期护理均由同一医疗团队提供。

VAS 评分(visual analogue scale，VAS)

0–10，0=无疼痛，1–3=轻度疼痛，4–6=中度疼痛，7–10=重度疼痛，

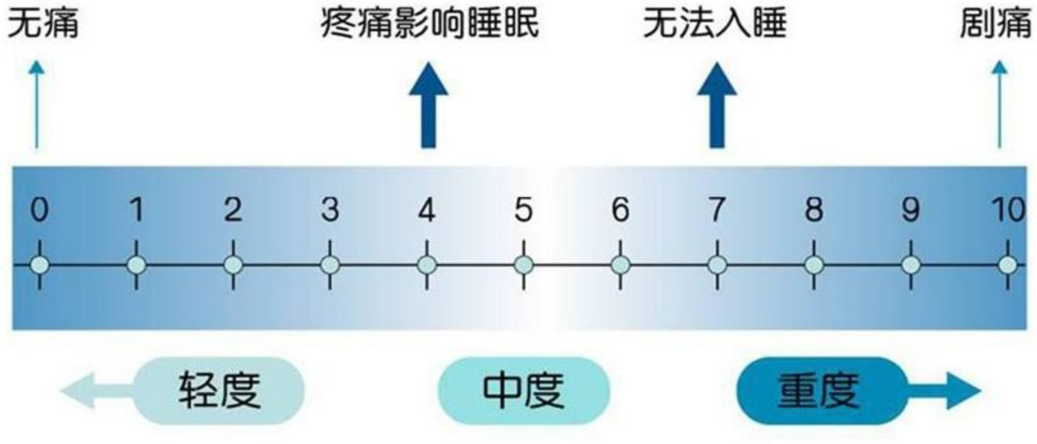

技术路线

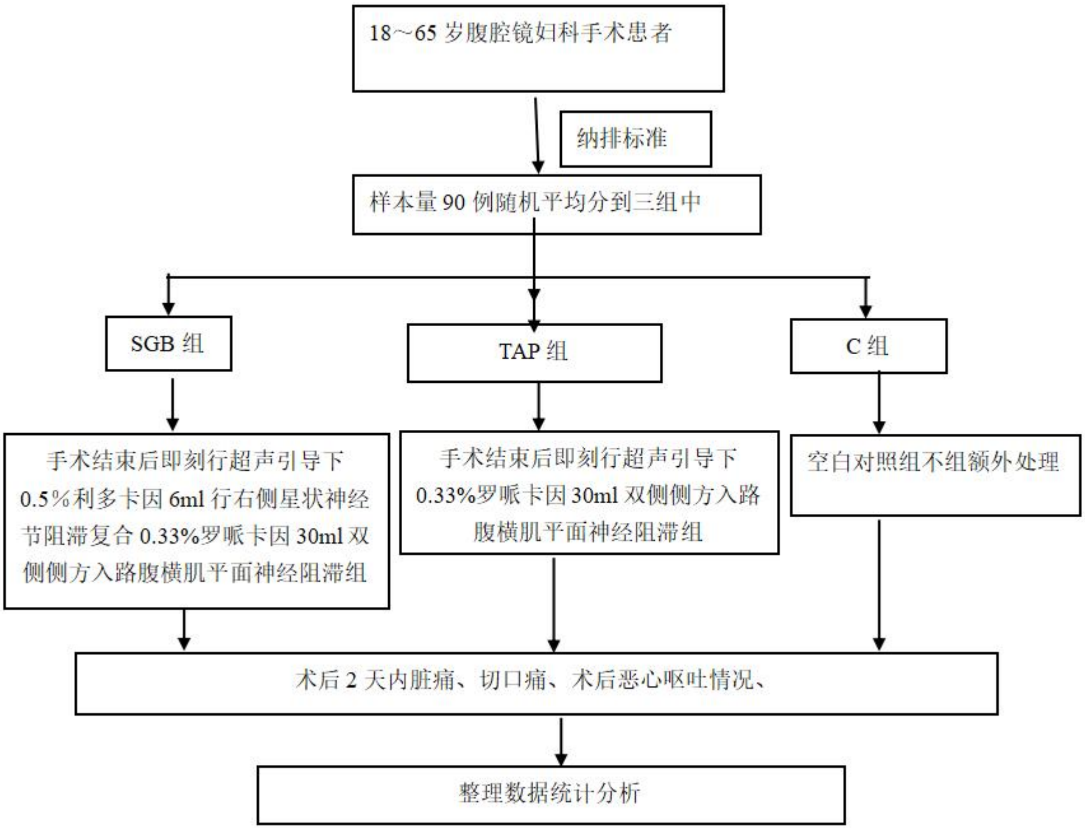
18～65 岁腹腔镜妇科手术患者
纳排标准
样本量 90 例随机平均分到三组中
SGB 组
TAP 组
C 组
手术结束后即刻行超声引导下 0.5%利多卡因 6ml 行右侧星状神经节阻滞复合 0.33%罗哌卡因 30ml 双侧侧方入路腹横肌平面神经阻滞组
手术结束后即刻行超声引导下 0.33%罗哌卡因 30ml 双侧侧方入路腹横肌平面神经阻滞组
空白对照组不组额外处理
术后 2 天内脏痛、切口痛、术后恶心呕吐情况、
整理数据统计分析
